# Cup revision involving retention of a fixed but malpositioned acetabular component in patients with poor general conditions

**DOI:** 10.1097/MD.0000000000008622

**Published:** 2017-11-17

**Authors:** Weiping Su, Min Zeng, Yihe Hu, Jianxi Zhu, Long Wang, Jie Xie

**Affiliations:** Department of Orthopedics, Xiangya Hospital, Central South University, Changsha, Hunan, China.

**Keywords:** acetabular component, malposition, revision, total hip arthroplasty

## Abstract

This study evaluated the surgical technique and outcomes of cup revision involving retention of a fixed but malpositioned acetabular component in patients with poor general conditions.

Between 2007 and 2013, we performed cup revision on 12 hips while retaining a fixed (either cemented or uncemented) but malpositioned acetabular component. Indications for this technique were: malpositioned but fixed acetabular shell; sufficient space for the insertion of the prosthesis; and patients with poor general conditions. After intraoperative confirmation of shell stability, a replacement liner was oriented in a new plane. Clinical and imaging data were collected perioperatively and during follow-up for evaluation of surgical efficacy.

No intraoperative complications were encountered. Mean operative duration was 70.4 minutes (range, 45–90 minutes) and mean estimated blood loss was 729 mL (range, 400–1200 mL). Mean follow-up duration was 5.1 years (range, 2.5–8.5 years). Average visual analog scale score decreased from (7.08 ± 1.00) preoperatively to (1.42 ± 0.67) at final follow-up (*P* < .05). Average Harris Hip Score improved from (14.7 ± 6.58) preoperatively to (80.9 ± 5.30) at final follow-up (*P* < .05). Anteversions and inclinations of new liners were (15.1 ± 2.3)° and (46.4 ± 3.9)° respectively. Postoperative radiographs showed no signs of prosthesis loosening, periprosthetic fractures, or dislocation compared with preoperatively.

The short-term efficacy of cup revision with retention of a malpositioned but fixed acetabular component was satisfactory.

## 1 Introduction

Although total hip arthroplasty (THA) is a successful surgical intervention for advanced joint disease, its efficacy can be compromised by component malpositioning and subsequent aseptic loosening.^[1]^ Cup revision is indicated for a malpositioned acetabular component,^[2,3]^ and many studies have demonstrated that, if the acetabular component to be revised is secure and well fixed, cementing a new liner into a pre-existing acetabular shell is a low-cost and effective treatment.^[4–9]^ However, when revision THA involves a fixed but malpositioned acetabular component, the decision-making process is much more difficult.^[2]^ The disadvantages of aggressive debridement and curettage are associated increases in blood loss; operative time and cost; loss of bone stock; and operative morbidity, especially with a cemented acetabular component.^[10]^As for patients with a poor general condition, the complexity of such an operation may lead to a fatal result.^[11,12]^ Therefore, because most patients with a poor general condition do not perform intensive exercise and require only a painless joint with basic functionality for daily activity, a simpler and less risky operation may be more appropriate. At our institution, for patients with a poor general condition, we perform the more conservative technique of cup revision with retention of a fixed but malpositioned acetabular component. The objective of the present study was to evaluate, through a retrospective review, the surgical technique and outcomes of this procedure in patients with poor general conditions.

## 2 Materials and methods

We performed a retrospective review of patients with a poor general condition who underwent cup revision involving retention of a fixed but malpositioned acetabular component at our institution from January 2007 to December 2013. The inclusion criteria were: the postoperative radiographic examinations of primary surgery showed malposition of the acetabular components which would undoubtedly lead to mechanical failure; the malpositioned acetabular shell was well fixed. Cups were deemed well-fixed if the preoperative radiographs were free of circumferential radiolucent lines at the bone–prosthesis interface, there was no evidence of component migration or screw breakage, and operative notes indicated that the cup was tested manually and there was no evidence of motion intraoperatively. Intraoperative examination showed that sufficient space was available for the insertion of the new prosthesis; patients were at levels of class III or class IV according to the American Society of Anesthesiologists (ASA) Physical Status classification system which means anesthesia risk of the surgery was high. The exclusion criteria were that the malpositioned acetabular component was caused by infection.

Twelve revision hip surgeries in 12 patients were identified. All clinical data were collected from the electronic medical records systems. Follow-up data were obtained from hospital charts face to face or by a phone call. For the patients we could not contact by telephone, we considered the most recent follow-up visit to be their final visit. All the patients were informed about this alternative surgery, and written consent of the surgery was obtained. The medical ethics committee of our institution approved the study.

### 2.1 Surgical technique

With patients under epidural anesthesia, a single senior surgeon performed all hip revisions via a posterolateral approach. The stability of the residual shell of uncemented cups was tested after liner removal and debridement of fibrous membranes. The stability of cemented acetabular components was tested after debridement. A shell was considered well fixed when no acetabular migration was observed after repeated, direct, maximum hand pressure on the edge of the component with a metal pusher. Hips with signs of possible movement underwent shell removal and complete debridement, and these patients were not included in the analysis. A reamer was used for acetabular reconstruction in cemented cups, and the debris from polyethylene wear was removed by repeatedly irrigating the joint. For uncemented cups, a burr was used to roughen the acetabular shell. Liners were trialed to determine the proper size, and at least 2 mm of space was left for the cement mantle.^[13]^ After cleaning and drying the inner surface of the socket, the cement, with a dough-like consistency, was inserted into the cavity under pressure and a new liner inserted. Because of the malposition of the residual shell, the new liner was oriented in a new plane with 5° to 25° of anteversion and 35° to 55° of inclination.^[3]^ The outside diameter of the liners after revision was 50 mm, and the femoral head was 28 mm in diameter. In addition, loosening on the femoral side had been ruled out in all patients on preoperative radiographs and intraoperative examinations, so revision of the femoral prosthesis was not required. After cementation and articular reduction, intraoperative hip stability was assessed by a range-of-motion test. A drain was placed, and the incision was sutured closed in layers.

Patients received cefazolin for 48 hours postoperatively, and rivaroxaban was begun postoperatively and continued for 2 weeks as prophylaxis against deep vein thrombosis. Drains were removed when there was no further increase in fluid drainage, usually 24 to 48 hours postoperatively. Patients were encouraged to mobilize in bed on postoperative day 1 and to walk with toe-touch weightbearing using crutches beginning after drain removal and continuing upon discharge. Ambulation with full weightbearing was permitted 3 months after surgery.

### 2.2 Evaluation

Clinical and radiographic data were collected preoperatively, postoperatively, and during the follow-up period (1, 3, 6, and 12 months postoperatively and every year thereafter). Clinical evaluation measures included operative time, bleeding volume in surgery, Harris Hip Score (HHS) (0–100 points; 100 = best function) and visual analog scale (VAS) score (0–10; 0 = no pain). Anteroposterior- and lateral-view radiographs of the hip were obtained to check the status of the prosthesis. Specifically, orientation of acetabular component, including anteversion and inclination, was measured.^[14]^ Acetabular osteolysis, periprosthetic radiolucency, component migration, and polyethylene wear were determined using approved criteria.^[15,16]^ The presence of a complete radiolucent line, component migration, or change in cup position suggested acetabular loosening.^[17]^

### 2.3 Statistical analysis

IBM SPSS Statistics for Windows, Version 19.0 (IBM Corp, Armonk, NY) was used for statistical analysis. Pre- and postoperative HHS and VAS scores were compared using paired *t* tests. A significant difference was defined as *P* < .05.

## 3 Results

Twelve revision hip surgeries (9 right, 3 left) in 12 patients (10 women, 2 men) were in our study. The average age at the time of index revision surgery was 75.4 years (range, 65–85 years). The initial THAs were all carried out at outside institutions. The average length of time from primary THA to revision surgery with cementation of liner (index surgery) was 1.62 months (range, 0–6 months). The primary acetabular shells were cemented in 9 hips and uncemented in 3 hips. Based on preoperative radiographic measurement, mean anteversions and inclinations of implanted liners were (32.4 ± 19.8)° and (34.7 ± 30)° respectively. Eight patients had an ASA class III and 4 patients had an ASA class IV according to ASA anesthesia risk assessment. And these patients had varying degree of pains. Revision hip surgery became a necessity in this setting, and cup revision was performed once diagnosed (Tables 1 and 2).

**Table 1:**
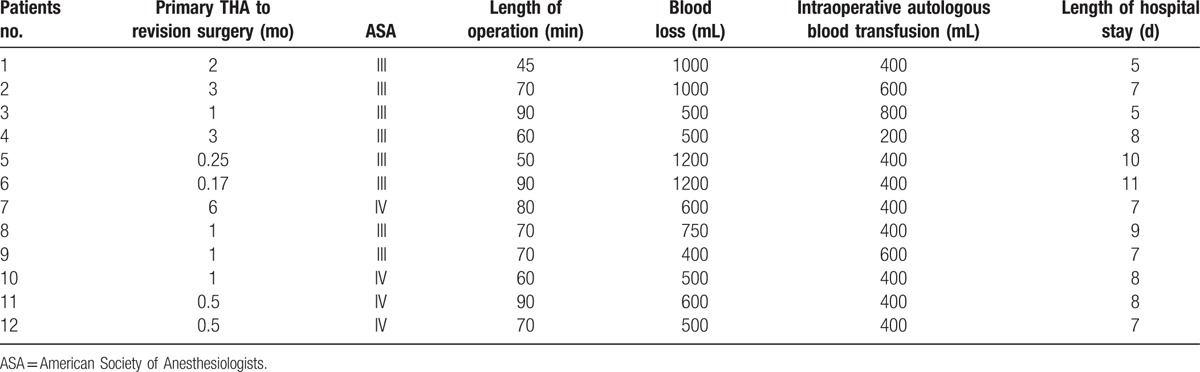
Patient characteristics.

**Table 2:**
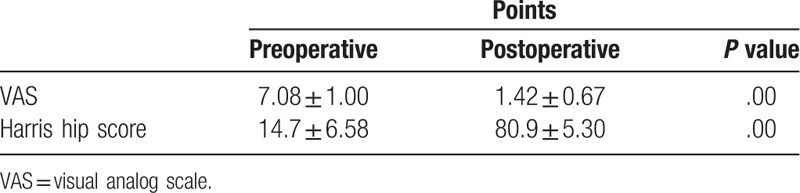
Patient outcomes.

**Figure 1:**
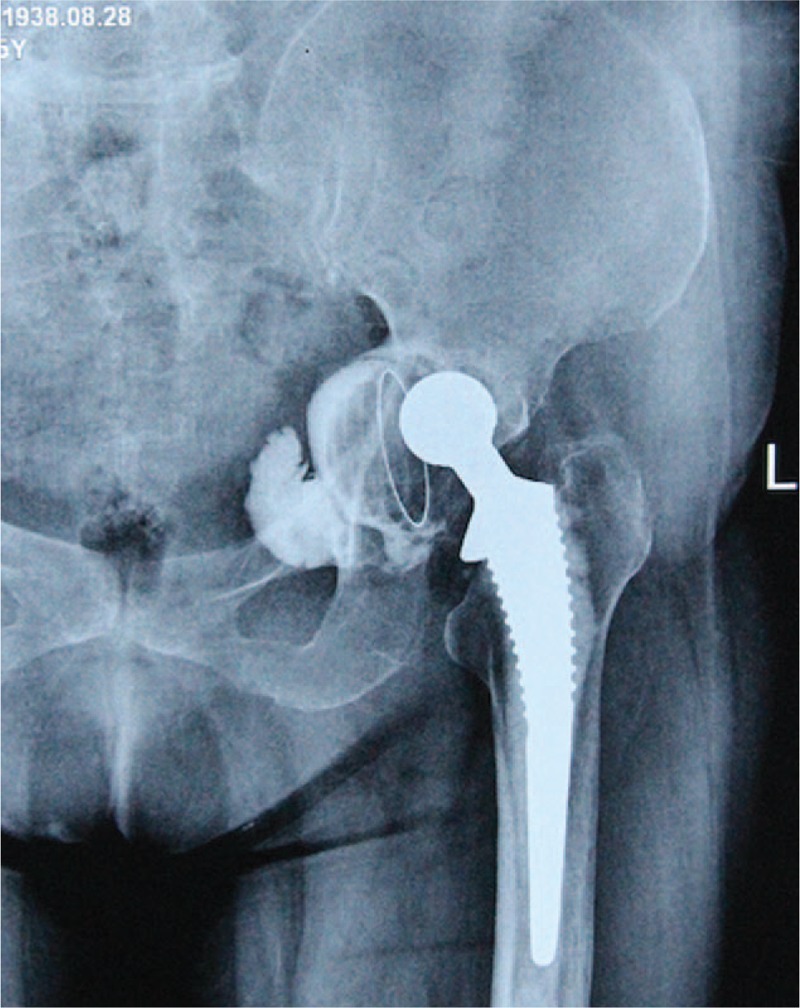
Preoperative anteroposterior radiographs of a 75-year-old female revealing a fixed but malpositioned acetabular component (cemented).

**Figure 2:**
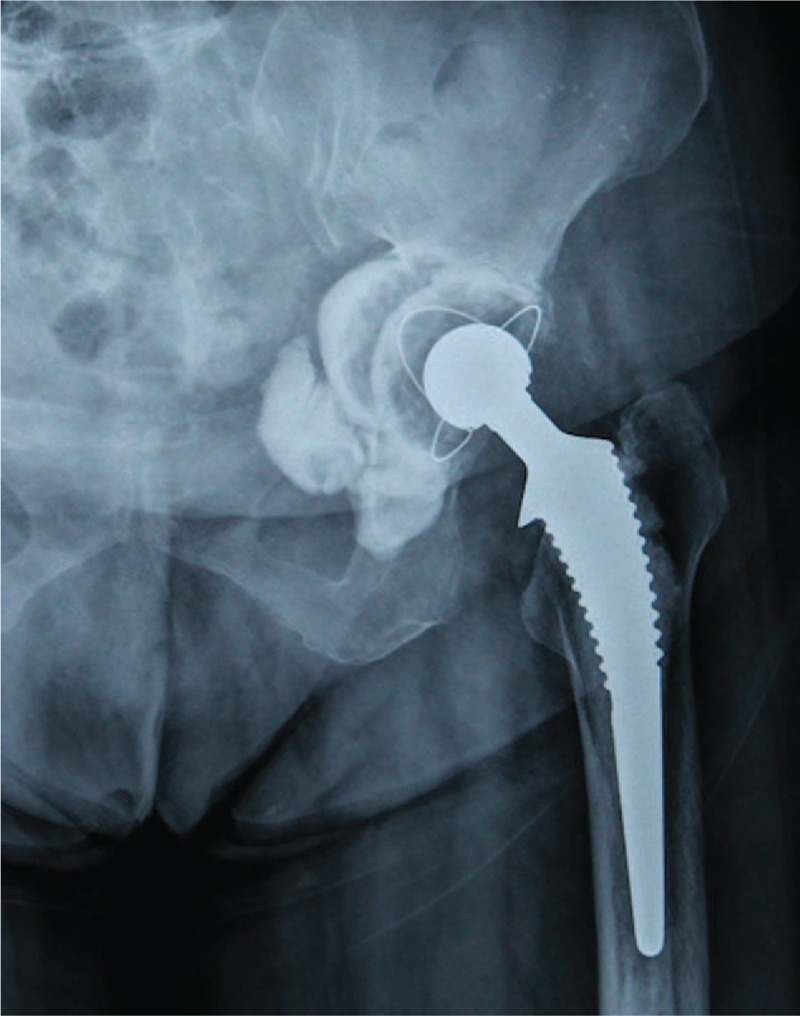
Postoperative anteroposterior radiographs of the patient after polyethylene-liner cementation revealing favorable state of the prosthesis.

**Figure 3:**
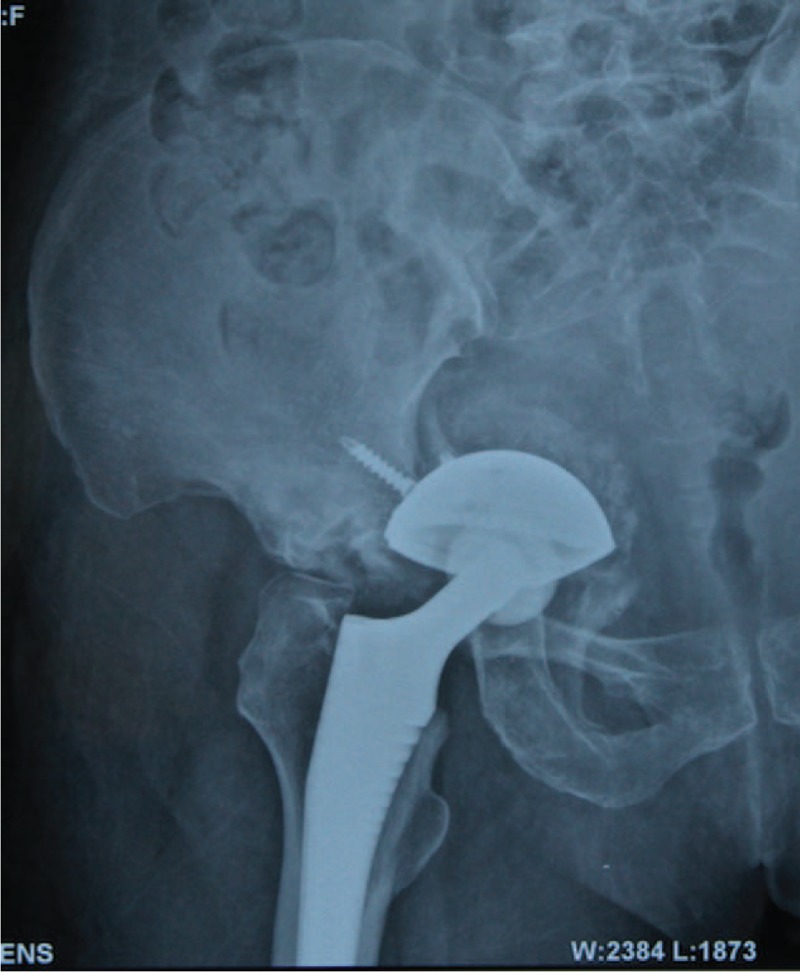
Preoperative anteroposterior radiographs of a 69-year-old female revealing a fixed but malpositioned acetabular component (uncemented).

**Figure 4:**
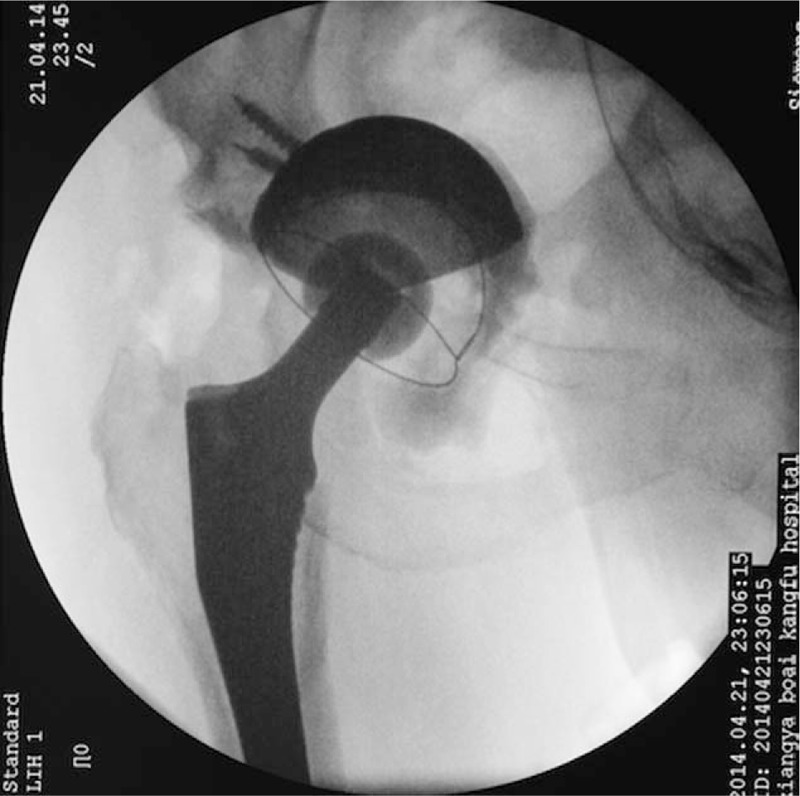
Intraoperative fluoroscopy of the patient revealing accurate positioning of the prosthesis.

**Figure 5:**
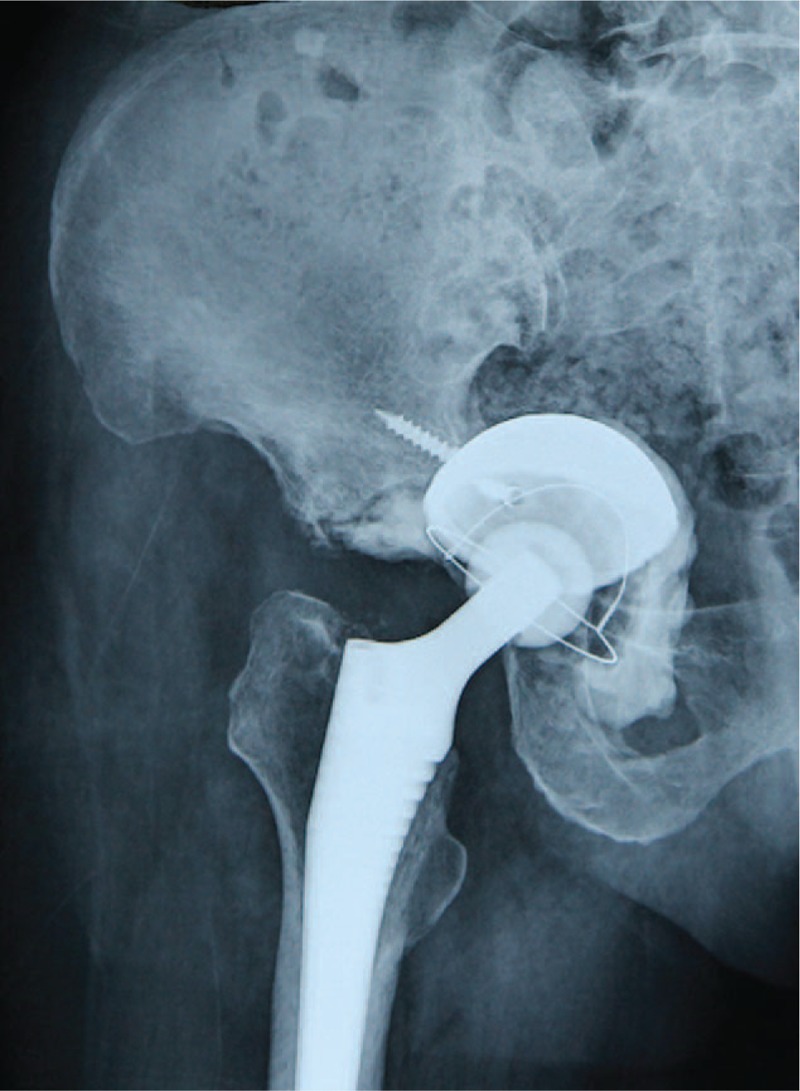
Postoperative anteroposterior radiographs of the patient after polyethylene-liner cementation revealing favorable state of the prosthesis.

All surgeries were conducted smoothly, without intraoperative complications. Mean operative duration was 70.4 minutes (range, 45–90 minutes) and mean estimated blood loss was 729 mL (range, 400–1200 mL). Mean intraoperative autologous blood transfusion was 450 mL (range, 200–800 mL). No additional homologous blood was required during hospitalization. The average hospital stay was 7.7 days (range, 5–11 days). Mean follow-up was 5.1 years (range, 2.5–8.5 years). Mean VAS score decreased significantly, from (7.08 ± 1.00) preoperatively to (1.42 ± 0.67) at final follow-up (*P* < .05). Mean HHS improved significantly, from (14.7 ± 6.58) preoperatively to (80.9 ± 5.30) at final follow-up (*P* < .05). Based on postoperative radiographic measurement, mean anteversions and inclinations of implanted liners were (15.1 ± 2.3)° and (46.4 ± 3.9)° respectively, indicating favorable prosthesis position. Comparison of preoperative and follow-up radiographs revealed no signs of prosthesis loosening, periprosthetic fracture, or dislocation (Figs. 1–5).

## 4 Discussion

Removal of a well-fixed acetabular component during revision THA can be extremely challenging, often causing associated increases in blood loss, operative time and cost, loss of bone stock, and operative morbidity.^[2,18]^ Liner cementation has been suggested for a well-fixed and well-positioned acetabular shell^[4–9]^; however, for a fixed but malpositioned acetabular component, aggressive cup revision may be not worth the effort, especially in patients with poor general condition. These patients usually suffer from many medical comorbidities, which affect surgical efficacy.^[10,19]^ At our institution, the decision to perform cup revision for a malpositioned acetabular component in patients who had American Society of Anesthesiologists Physical Status classification system class III or IV is made on the basis of the pre-existing acetabular component. Our intraoperative data show that this simple technique results in less injury and fewer complications. Operative duration and blood loss were decreased, which is extremely important in patients with poor general condition. Our clinical data, including HHS and VAS score, demonstrated satisfactory pain relief and functional improvement.

Dislocation, a common postoperative complication of hip revision, can result from repeated soft tissue disruption, different types of prostheses, and poor prosthesis orientation.^[9,20]^ The use of a relatively smaller head size and inaccurate acetabular orientation during revision with retention of the acetabular component have led to a rise in the occurrence of hip dislocation.^[2,4,6]^ The femoral heads were 28 mm in diameter in our study. No patient in the present study experienced dislocation, which was likely due to both the lower activity levels of the high-risk population and the minimal invasiveness of this technique. Also, all stem prostheses were securely fixed, and no femoral revision was required. The new liner was oriented in a plane with 5° to 25 of anteversion and 35° to 55° of inclination, and intraoperative fluoroscopy was used to ensure accurate positioning of the prosthesis.

In 1 study of 100 hips treated with revision THA with a retained acetabular component, the failure rate was 13% over a mean follow-up of 6.6 years.^[8]^ Another study of 31 hips reported that, other than 2 hip dislocations, both treated conservatively, no major complications occurred over a mean follow-up of 5.3 years.^[7]^ Finally, Talmo et al^[6]^ reported rerevision rates of 15% following full acetabular revision of a well-fixed cup and 27% following acetabular revision with retention of a fixed cup. The results of the present study, including clinical data and radiographs, demonstrated favorable status of prostheses. The variation in these results may have resulted from differences in patient populations, surgical skill, and postoperative rehabilitation. The absence of failures in our cohort was largely due to the strict criteria for selection of cases; that is, a fixed acetabular shell and enough space left for the insertion of a new prosthesis, and high-risk patients. Instability of the acetabular component is a risk factor for failed revision.^[4,5,21]^ A complete acetabular revision should be considered when signs of possible instability are seen on preoperative radiographs and intraoperative examination. According to previous studies,^[9,13]^ the cement mantle should be at least 2 mm thick. For primary cemented THA, copious irrigation was performed multiple times during acetabular reconstruction to remove the debris resulting from polyethylene wear. We used a highly cross-linked polyethylene liner for cup revision because of the ability of that material to halt osteolysis.^[22]^ After surgery, the patients were advised to walk with crutches for the first 3 months.

The disadvantage of this technique is the creation of 2 more interfaces, one between the retained cup component and cement and the other between the cement and new acetabular component, which increases the likelihood of prosthesis loosening. There is also a hidden danger to prosthetic stability from recurrence of loosening of the fixed primary acetabular component, and rerevision in this setting is extremely difficult.

The present study had some limitations. First, the number of patients was small, but clinical and radiographic data were collected prospectively with no defaulter. Second, the follow-up period was relatively short, but a mean of 5.1 years was sufficient to detect the short-term effects of the procedure. However, our findings should be further validated by larger, well-powered, prospective studies.

## 5 Conclusion

The short-term efficacy of cup revision with retention of a fixed but malpositioned acetabular component was satisfactory.
